# Clinico-Epidemiological Analysis of Small Bowel Obstruction in Adults at a Tertiary Care Center in India

**DOI:** 10.7759/cureus.63278

**Published:** 2024-06-27

**Authors:** Shubham Rani, Ashok Puranik, Ramkaran Chaudhary, Poonam Elhence, Taruna Yadav, Vaibhav K Varshney

**Affiliations:** 1 General Surgery, All India Institute of Medical Sciences, Jodhpur, Jodhpur, IND; 2 Pathology, All India Institute of Medical Sciences, Jodhpur, Jodhpur, IND; 3 Radiology, All India Institute of Medical Sciences, Jodhpur, Jodhpur, IND; 4 Surgical Gastroenterology, All India Institute of Medical Sciences, Jodhpur, Jodhpur, IND

**Keywords:** intestinal stricture, explorative laparotomy, ileum obstruction, small bowel surgery, adhesive small bowel obstruction

## Abstract

Background: Acute small bowel obstruction (SBO) is a common surgical emergency. The study aims to provide a comprehensive clinical-epidemiological description of SBO in adults at a tertiary care center in western India.

Methods: This hospital-based cross-sectional study was conducted from July 2020 to June 2022 and enrolled 88 SBO patients requiring surgical intervention. After adequately resuscitating the patients, various surgical procedures were performed based on the intraoperative conditions of the bowel. Patients were assessed postoperatively for the duration of their hospital stay, postoperative complications, and surgical recovery.

Results: There was a male preponderance (n=55), with a median age of 50 (18-90) years. Abdominal discomfort was the most frequent symptom, necessitating a hospital visit (97.9%, n= 86), followed by nausea (85.2%, n= 75), constipation (78.1%, n=69), and abdominal distension (51.1%, n=45). Ileal strictures (18.2%, n=16) were the most common etiology, followed by postoperative adhesions (14.8%, n=13) and bands (13.6%, n=12), of which 76.4% (n=9) had past surgical history. Resection and anastomosis were the most frequently performed surgical interventions in this study (36.4%, n=32), followed by stoma creation (27.3%, n=24) and adhesiolysis (17%, n=15). The postoperative 30-day mortality of 11.36% (n=10) was noted, which could be ascribed to the elderly population with comorbidity, postoperative complications, and who required extended stay in the critical care unit.

Conclusion: Benign ileal stricture was the most common cause of acute SBO in the emergency. Prompt and timely diagnosis combined with a multidisciplinary approach and effective management can improve outcomes and reduce morbidity and mortality in adult patients with SBO.

## Introduction

Small bowel obstruction (SBO) is a common surgical emergency worldwide [1]. It is a reason for 12-16% of surgical hospitalizations for acute abdominal pain [2]. The mechanical cause of intestinal obstruction is a significant component of diseases requiring emergency surgical procedures in Asia, including India [3,4]. It is a potentially dangerous surgical emergency that can result in substantial morbidity and mortality [5,6]. Initially, all patients with intestinal obstruction are managed with conservative management, such as fluid resuscitation, vital monitoring, antibiotics, and nasogastric drainage. Among them, a few respond and convert to sub-acute intestinal obstruction and can be operated in elective settings. The rest of the patients with acute SBO require surgical interventions in an emergency.

Although the mortality rate from acute SBO is lower in urban areas due to early presentation and timely and appropriate medical care, it is not the case in rural areas due to delayed presentation leading to various complications [3]. Inadequate clinical judgment is one of the reasons that leads to a poor prognosis in cases of SBO [7]. In 80% of instances, intestinal obstruction affects the small bowel, and 20% impacts the large intestine [1].

In different countries, acute intestinal obstruction has a range of causes, and the causes have evolved over time [8]. Though there are a variety of causes for this ailment, intra-abdominal adhesions from earlier abdominal surgery are the etiologic factor in up to 75% of small intestinal obstruction cases [9,10]. Apart from adhesions, the other notable causes are malignancy (15%), inflammatory strictures (15%), obstructed hernia (12%), fecal impaction (8%), and pseudo-obstruction (1%). Throughout the previous century, the small bowel has been more commonly involved in causing intestinal obstruction than the large bowel; however, the etiological variables in small and large bowel obstruction have changed dramatically [11].

This study aimed to provide a complete epidemiological description of SBO in adults in a tertiary care center. The primary objective is to determine the etiology of SBO in adults, with secondary objectives to assess postoperative outcomes like surgical site infection, septicemia, wound dehiscence, enterocutaneous fistula, and anastomotic leak affecting morbidity and mortality in this population.

## Materials and methods

This is a hospital-based descriptive study in which all patients diagnosed with SBO who met the inclusion criteria were enrolled in the Department of General Surgery at All India Institute of Medical Sciences, Jodhpur, India. After getting the All India Institute of Medical Sciences, Jodhpur Ethics Committee approval (AIIMS/JDH/2020/3176), the data was prospectively maintained from July 2020 to June 2022. Written and informed consent from the patient or next of kin was obtained.

The inclusion criteria are adult patients over 18 years of age and patients who had SBO based on clinical and radiological parameters.

The exclusion criteria are subacute intestinal obstruction managed conservatively and pseudo-obstruction, which is defined as dilation of the bowel in the absence of an anatomical obstruction.

Perioperative management

All patients with clinical features suggestive of SBO were resuscitated with intravenous fluids, antibiotics, analgesics, and antiemetics, along with the placement of a nasogastric tube and urinary catheter. They were subjected to blood tests such as a hemogram, renal and hepatic function test, serum electrolytes, and arterial blood gas analysis, among other measures. Initially, to ascertain the diagnosis of SBO, an abdominal radiograph in erect and supine positions was taken. Further, a contrast-enhanced computed tomography of the abdomen was conducted to determine the exact cause of the obstruction unless there were contraindications for it to be done, such as renal impairment.

After the pre-anesthetic check-up, as part of surgical site prophylaxis, all patients received an intravenous Ceftriaxone 1 g 30 minutes before the skin incision. Based on the intraoperative findings, an operative diagnosis is established. In the postoperative period, all patients received intravenous antibiotics and adequate analgesics. The patient was mobilized adequately on the first postoperative day, and incentive spirometry was implemented. Following the passage of flatus or when the stoma got functional, patients were permitted to consume orally, beginning with sips of water and progressing to a complete oral meal.

Factors like intra-operative findings, length of hospital stay, the timing of resuming oral feeds, operative and postoperative complications, mortality, and morbidity according to the Clavien Dindo classification were assessed. The relationship between the preliminary diagnosis based on the CT scan, the intraoperative diagnosis, and the etiology of the SBO based on histopathology was established.

Outcome parameters

Preoperative data on the patient’s demographics, symptomatology, co-morbidities, prior surgery history, and investigations were gathered. Intraoperative findings, procedures performed, and complications were documented. For each patient, postoperative data such as the timing of the passage of flatus, the timing of the initiation of oral feed, and the incidence of surgical site infection were also gathered.

Statistical analysis

Data was entered in Microsoft Excel software (Microsoft Corporation, Redmond, United States). Categorical data was presented as frequency and percentage. Continuous data was expressed as a median and interquartile range.

## Results

Out of 88 patients recruited in the study, 55 were males (62.5%), with the median age of patients being 50 (18-90) years. Pain in the abdomen was the most common presenting symptom in 86 patients (97.7%), followed by vomiting (85.2%, n=75), obstipation (78.4%, n=69), and distention (51.1%, n=45). Among patients with comorbidities, 11 patients were hypertensive (12.5%), 4 had COPD (4.5%), and three were diabetic (3.4%). Forty patients (45.5%) had a history of previous abdominal surgery, and 31.8% (n=28) of patients were hospitalized with similar complaints in the past and were treated conservatively. On assessment, 52 individuals (59%) had exaggerated (>30 sounds per minute on auscultation), 8 (9.1%) had normal (5-30 sounds per minute), and 28 (31.9%) had hypoactive (less than 5 sounds per minute) bowel sounds (Table 1).

**Table 1:** Demographic profile and clinical features COPD: chronic obstructive pulmonary disease

Parameters	Number of patients (n)	Proportion of patients (%)
Gender		
Male	55	62.5
Female	33	37.5
Age group (years)		
18-40	27	30.7
41-60	38	40.6
>60	23	26.1
Presenting complaints		
Pain abdomen	86	97.7
Vomiting	75	85.2
Obstipation	69	78.4
Distention	45	51.1
Past medical history		
History of similar episodes	28	31.8
History of surgical intervention	40	45.5
Co-morbidities		
Hypertension	11	12.5
Tuberculosis	6	6.8
Coronary artery disease	5	5.7
COPD	4	4.5
Diabetes mellitus	3	3.4
Chronic kidney disease	1	1.1
Others	14	
Addictions		
Alcohol	12	13.6
Smoking	17	19.3
Tobacco	12	13.8
Opium	12	13.6
Abdominal examination findings		
Distension	72	81.8
Tenderness	58	65.9
Previous surgical scars	40	45.5
Ballooning empty rectum	38	43.2

On erect abdominal radiographs, 59.1% (n=52) of patients had multiple air-fluid levels in the small bowel and dilated loops of the small bowel (>5 cm) were noted in 40.9% (n=36) of the patients. However, none of them had free air under the diaphragm. It should be noted that the diagnostic accuracy of the CT scan was 93.2% when this was corroborated intra-operatively. However, clinical assessment should be applied in diagnosis wherever possible, and higher investigations such as CT scans should be used only whenever necessary (Table 2).

**Table 2:** Radiological investigations

Investigations	Number of patients (n)	Percentage of patients (%)
Erect abdominal radiograph		
Multiple air-fluid levels	52	59.1
Dilated bowel loops	36	40.9
Computed tomography		
Transition point	55	63.9
No transition point	31	36.04

The median duration of the time interval between admission and surgery was 12 (1-456) hours, the median postoperative stay was 8 (1-52) days, and the median length of hospital stay was 9 (1-52) days. The most common etiology encountered was ileal strictures (18.2%, n=16), followed by postoperative adhesions (14.8%, n=13) and bands (13.6%, n=12). Among the ileal strictures, eight were inflammatory in nature on histopathology, four were ischemic, and two each were infective and hyperplastic. About 17 patients had adhesions as etiology, of which 13 had past surgical history. Paracecal (n=2) and transmesenteric (n=2) were the common internal hernia encountered, and the femoral hernia (n=3) was the most common external hernia noticed. Resection and anastomosis were the most common procedures (36.4%, n=32), followed by stoma formation without anastomosis (27.3%, n=24) and adhesiolysis (17%, n=15). Further, histopathological examination of these resected specimens showed non-specific findings in 15 patients (46.8%), hyperplasia, malignancy, and vasculitis in 5 patients (15.6%) each, and features of tuberculosis in 2 patients (6.2%). Small bowel adenocarcinoma was the most common malignancy leading to SBO, followed by neuroendocrine tumors and metastatic lesions, which were the other etiologies (Table 3).

**Table 3:** Various etiologies and procedures done

	Number of patients (n)	Percentage of patients (%)
Etiology		
Ileal stricture	16	18.2
Postoperative adhesions	13	14.8
Bands	12	13.6
Internal hernia	09	10.2
Abdominal wall hernias	09	10.2
Neoplasm	08	9.1
Adhesions	04	4.5
Kochs abdomen	04	4.5
Acute mesenteric ischemia	03	3.4
Fecal impaction	03	3.4
Midgut volvulus	02	2.3
Intussusception	02	2.3
Appendicular perforation	01	1.1
Meckel’s diverticulitis	01	1.1
Mucormycosis	01	1.1
Procedures done		
Resection and anastomosis	32	36.4
Stoma formation	24	27.3
Adhesiolysis	15	17
Band release	10	11.4
Reduction of hernial contents with herniorraphy	07	7.9

Among the postoperative complications, 27.27% (n=24) suffered superficial surgical site infection followed by burst abdomen (10.23%, n=9), shock (7.95%, n=7), septicemia (4.55%, n=4), wound dehiscence (3.41%, n=3), anastomotic leak (2.27%, n=2), and enterocutaneous fistula (1.14%, n=1). As per the Clavien-Dindo classification of complications, 12.5% (n=11) of patients were in Grade I, 8% (n=7) in Grade II and Grade III, 1.1% (n=2) in Grade IV, and 11.36% (n=10) in Grade V. Out of the total patients, 10 suffered from mortality, cause being refractory septic shock as most common followed by aspiration pneumonitis (respiratory failure) and refractory hypotensive shock (Table 4).

**Table 4:** Postoperative complications

	Number of patients (n)	Percentage of patients (%)
Complications		
SSI (superficial)	24	27.27
Burst abdomen	09	10.23
Shock	07	7.95
Septicemia	04	4.55
Wound dehiscence	03	3.41
Anastomotic leak	02	2.27
Enterocutaneous fistula	01	1.14
Clavien-Dindo classification		
< Grade III	25	28.5
≥ Grade III	12	12.46
Cause of death		
Refractory septic shock	07	70
Aspiration pneumonitis	02	20
Refractory hypotensive shock	01	10

## Discussion

SBO is a common surgical emergency whose management strategies have changed over the years. Acute intestinal obstruction can be caused by various factors, including adhesion, hernia, and cancer, as well as less common conditions such as intussusception [12]. In our study, the majority of the patients were male (62.5%), like other studies [7,12]. Several factors may contribute to this phenomenon, including anatomical variations, hormonal influences, and gender-specific risk factors for underlying causative conditions. For instance, the increased prevalence of inguinal and femoral hernias in males may partially explain the higher incidence of obstructive events in this gender. Further, the mean age of presentation was 48.5 years, similar to many previous studies [12,13]. The variations in mean age reported across different studies likely reflect the interplay of geographic, environmental, and demographic factors influencing the epidemiology of intestinal obstruction.

The most common symptom requiring a hospital visit was abdominal pain (97.9%), followed by vomiting (85.2%), constipation (78.1%), and abdominal distension (51.1%). These findings are inconsistent with those of Adhikari et al. [8]. On examination, most patients (81.8%) had distension, followed by tenderness (65.9%) and a pre-existing scar (45.5%), which is similar to another study done by Duron et al [14]. It was observed that patients with features of peritonitis at presentation had higher rates of complications and mortality. However, it is noteworthy that the absence or atypical presentation of these symptoms should not preclude further evaluation, as some cases may present with subtle or non-specific findings, particularly in the elderly or immunocompromised population.

The past medical history is invaluable in identifying the etiology of the present state. Out of four patients who were diagnosed with Koch’s abdomen, two had a history of tuberculosis. Forty patients had a history of abdominal surgery, out of which 13 patients had postoperative adhesions as the cause of obstruction. This finding also underscores the importance of adhering to meticulous surgical techniques, employing adjunctive measures to minimize adhesion formation, and carefully considering the risks and benefits of elective abdominal procedures, especially in individuals with a history of multiple abdominal surgeries.

The diagnostic accuracy of CT scans was 93.2%, indicating the significance of CT scans as an axillary tool for preoperative planning. Eighty-six patients in our study underwent CECT abdomen, of which only six patients were there whose CT scans were inconsistent with intraoperative findings (6.98% of patients). Suri et al. also mentioned that CT has high sensitivity (93%), specificity (100%), and accuracy (94%) in detecting the existence of obstruction [15].

The most common etiology in our study was ileal strictures (18.2%), followed by surgical adhesions (14.8%) and bands (13.6%). Our finding contrasts the notion that postoperative adhesions are the most common etiological factor for SBO [3]. Further, on histological examination, most of the ileal strictures were inflammatory, and the rest were infective, ischemic, and hyperplastic. This could be owing to the difference in the environment and living conditions in the western part of India and the prevalence of gastrointestinal pathologies, such as inflammatory bowel diseases or infectious etiologies, which can lead to stricture formation. Most of our patients underwent resection and anastomosis (36.4%), followed by stoma creation (27.3%) and adhesiolysis (15%). The open approach was predominant (65.9%), followed by laparoscopy-assisted (26.1%) and laparoscopy alone in 8% of patients. The underlying etiology often dictates the choice of procedure, the degree of bowel compromise, and the patient's overall clinical status.

The incidence of postoperative morbidity was noticed in 56.82% of patients. Potential contributing factors were advanced age, comorbidities, and the development of postoperative complications. This emphasizes improvement in patient selection, preoperative optimization, and postoperative care. Over one-third of patients were managed with regular wound care and one-fourth with the upgrade of antibiotics. However, less than one-third of patients required mesh laparostomy for burst abdomen. The median hospital stay was proportionally increased with the severity of complications.

The mortality rate in our study was 11.36%, and the most common reason was septic shock followed by anastomotic leak-related wound dehiscence. It was slightly higher than the previously reported series [12,13]. Prompt resuscitation, broad-spectrum antibiotic therapy, source control through surgical intervention, and supportive care in an intensive care setting are essential components of sepsis management. Additionally, efforts should be directed toward identifying and addressing potential modifiable risk factors that may predispose patients to septic complications in this clinical setting. Patients with comorbidities faced a 70% mortality rate, surpassing the 30% rate for those without.

Further, those aged ≤60 years exhibited a 40% mortality rate, in contrast to 60% for those who were above 60 years. Underlying medical conditions, such as diabetes, cardiovascular disease, or immunocompromised states, can adversely impact the body's ability to tolerate the physiological stress of surgery and cope with postoperative challenges. This finding underscores the importance of a multidisciplinary approach involving preoperative risk stratification, optimization of comorbidities, and close postoperative monitoring and management of high-risk patients.

Despite being a prospective study, the small sample size is one of the limitations of the study. A multicentric study with a large sample size can further confirm our observation of ileal stricture being a more common cause of SBO. Apart from this, the exclusion of non-operatively managed patients, whose inclusion would have allowed for a more comprehensive and comparative analysis.

## Conclusions

Inflammatory ileal strictures were the primary cause of intestinal obstruction, followed by postoperative adhesions and bands, with increased incidence in older adults. Early diagnosis through clinical examination, vital signs, and CT scans is crucial to prevent complications like gut gangrene. While conservative treatment may be suitable for adhesions, surgery is often required due to potential complications. Older patients with comorbidities and features of septic shock at presentation faced higher postoperative complications and mortality rates. A multidisciplinary approach involving departments like emergency medicine, diagnostic and intervention radiology, surgical gastroenterology, anesthesiology, and intensive care unit, apart from general surgery, are required to provide comprehensive care to enhance patient outcomes in SBO. Finally, aggressive management is essential for favorable outcomes.
